# Supplementary material for: A subset of type-II collagen-binding antibodies prevents experimental arthritis by inhibiting FCGR3 signaling in neutrophils
Source: Nat Commun. 2023 Sep 23;14:5949. doi: 10.1038/s41467-023-41561-7 (PMC10517938; doi:10.1038/s41467-023-41561-7)
Supplement: Supplementary file 1 — Supplementary Information [file 41467_2023_41561_MOESM1_ESM.pdf]

Supplementary materials

A subset of type-II collagen-binding antibodies prevents experimental arthritis by inhibiting FCGR3 signaling in neutrophils

Zhongwei Xu[1], Bingze Xu[1], Susanna L. Lundström[2], Àlex Moreno-Giró[1,3], Danxia Zhao[1], Myriam Martin[4], Erik Lönnblom[1], Qixing Li[5], Alexander Krämer[1], Changrong Ge[1], Lei Cheng[1], Bibo Liang[1,5], Dongmei Tong[1], Roma Stawikowska[6], Anna M. Blom[4], Gregg B. Fields[6], Roman A. Zubarev[2], Rikard Holmdahl*[1]

[1] Division of Medical Inflammation Research, Department of Medical Biochemistry and Biophysics, Karolinska Institute, Stockholm, Sweden

[2] Division of Physiological Chemistry I, Department of Medical Biochemistry and Biophysics, Karolinska Institute, Stockholm, Sweden

[3] Redoxis AB, Lund, Sweden

[4] Department of Translational Medicine, Lund University, Malmö, Sweden

[5] Center for Medical Immunopharmacology Research, Southern Medical University, Guangzhou, China

[6] Institute for Human Health & Disease Intervention and Department of Chemistry & Biochemistry, Florida Atlantic University, Jupiter, Florida, United States of America

* **Correspondence to:** Prof. Rikard Holmdahl, MD, PhD. Email: rikard.holmdahl@ki.se

Table of contents

- 1. Supplementary figures
- 2. Supplementary tables
- 3. Supplementary notes

1. Supplementary figures

Figure. S1

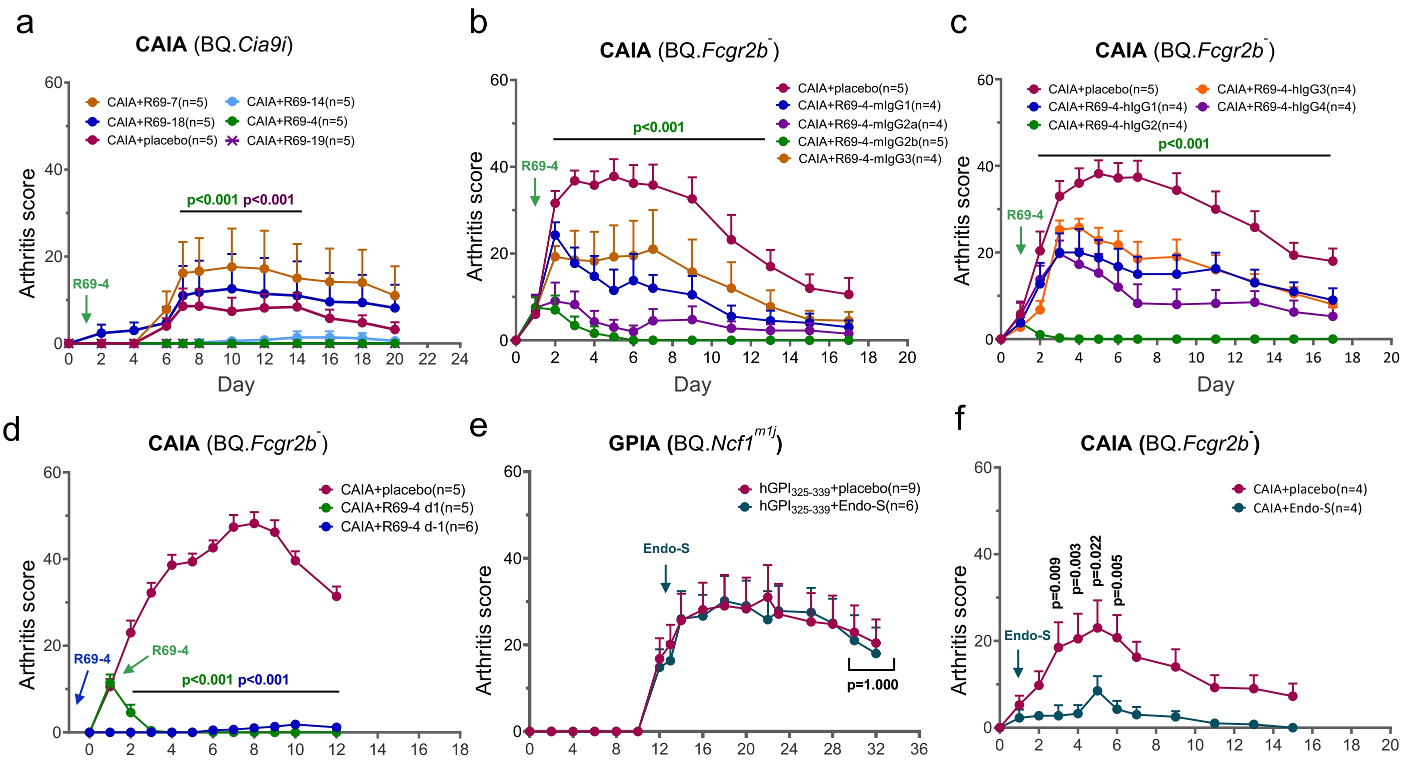

Fig S1. **a**. Screening of recombinant antibodies in cartilage antibody induced arthritis (CAIA) model (BQ.*Cia9i*). Cartilage antibody cocktail (M2139 and CIIC1): 9 mg, d0, *i.v.*; R69 series: 4.5 mg, d1, *i.v.*; lipopolysaccharide (LPS) boost: 50 µg, d4, *i.p.*; R69-4 and R69-19 protected against arthritis efficiently (Two-way ANOVA, p values marked for post-hoc testing of placebo *vs* R69-4 (green), or R69-19 (purple)). **b**. Screening of R69-4 isotypes in CAIA (BQ.*Fcgr2b*$^-$). Cartilage antibody cocktail (Cab4): 2 mg, d0, *i.v.*; R69-4 isotypes: 1 mg, d1, *i.v.*; R69-4 mIgG2b showed the best protection against arthritis (Two-way ANOVA, mIgG2b *vs* placebo: p<0.001 from d2 to d13). **c**. R69-4 with human isotypes protected against CAIA in *Fcgr2b*$^-$ mice. Cab4: 2 mg, d0, *i.v.*; human R69-4 isotypes: 1 mg, d1, *i.v.*; Human IgG2 isotype showed the best efficiency against arthritis (Two-way ANOVA, hIgG2 *vs* placebo: p<0.001 from d2 to d17). **d**. Efficacy of R69-4 in preventing CAIA (BQ.*Fcgr2b*$^-$). Pre-injection of R69-4 (d-1) prevented CAIA onset, and post-injection of R69-4 (d1) eliminated inflammation rapidly. Cab4: 2 mg, d0, *i.v.*; R69-4 mIgG2b: 1 mg, d-1 or d1, *i.v.* Two-way ANOVA, p values marked for post-hoc testing of placebo *vs* R69-4 d1 (green), or R69-4 d-1 (blue)); **e**. Efficacy of Endo-S in hGPI$_{325-339}$ peptide induced arthritis (GPIA) in BQ.*Ncf1*^{m1j} mice. hGPI$_{325-339}$ peptide: 10 µg emulsified in CFA, d0, *i.d.*; Endo-S: 25 µg, d13, *i.v.*; No significant difference was detected between the Endo-S group and the PBS group throughout the followed-up disease phase (Two-way ANOVA); **f**. Efficacy of Endo-S in CAIA (BQ.*Fcgr2b*$^-$). Cab4: 2 mg, d0, *i.v.*; Endo-S: 25 µg, d1, *i.v.*; Endo-S injection ameliorated antibody induced inflammation (Two-way ANOVA). All data shown in mean + standard error of mean (SEM). Placebo: PBS in a, e, and f; 0.01 M glycine + 0.15 M NaCl in b-d.

Figure. S2

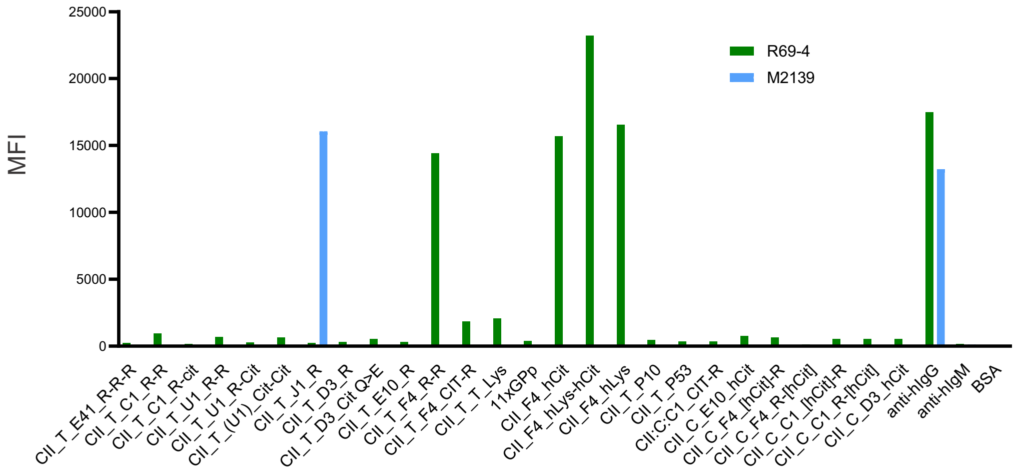

Fig S2. Specificity and cross-reactivity of R69-4 hIgG2 to common COL2 epitopes. R69-4 showed the highest binding to the F4 epitope and mild binding to multiple other epitopes, whereas human M2139 showed binding to J1 epitope exclusively. Antibody concentration: 100 ng/mL. MFI: median fluorescent intensity.

Figure. S3

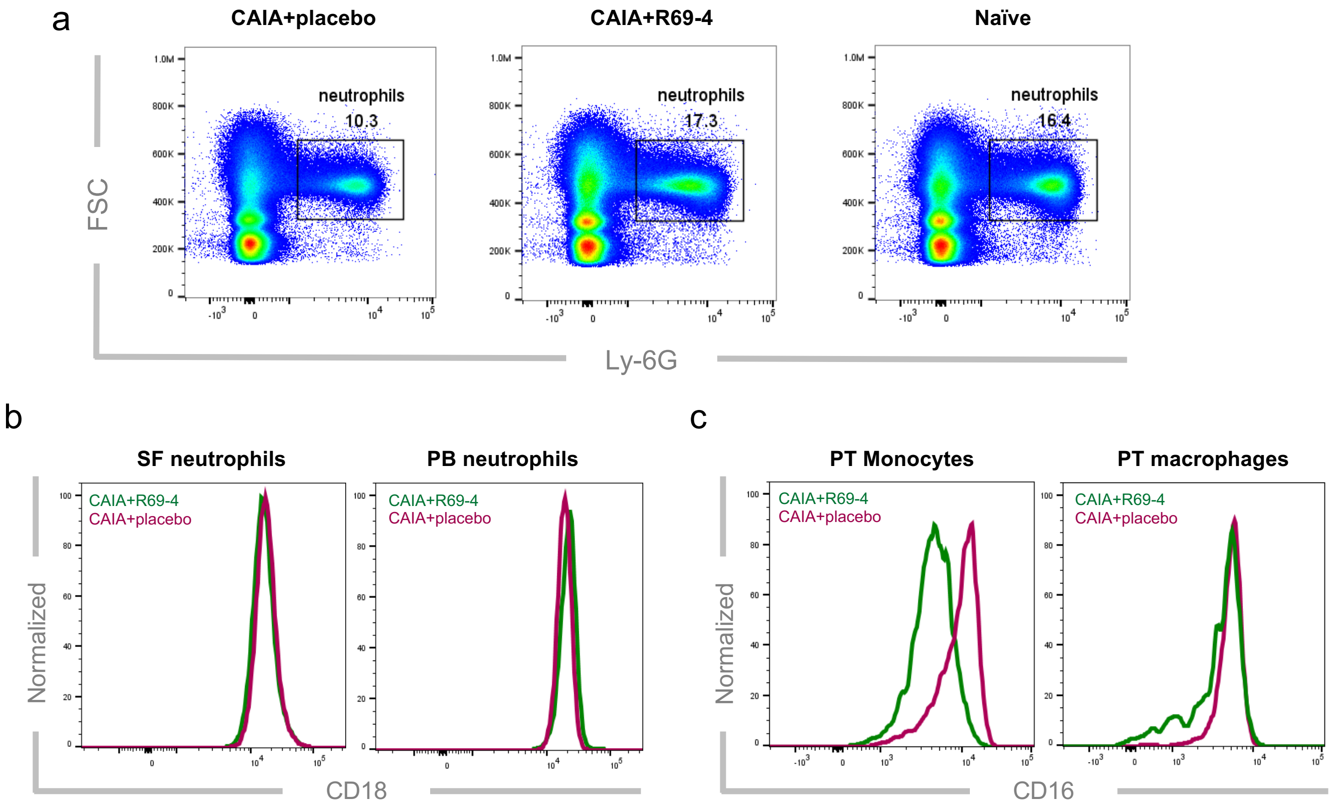

Fig S3. **a**. Representative plots showing that R69-4 injection restricted more neutrophils within bone marrow after CAIA onset. **b**. Representative plots showing that R69-4 injection did not alter the expression of CD18 on neutrophils from either synovial fluid (SF) or peripheral blood (PB). **c**. Representative plots showing that R69-4 injection down-regulated the expression of FCGR3 (CD16) on monocytes from pannus tissue (PT), whereas the effect was mild on PT macrophages.

Figure. S4

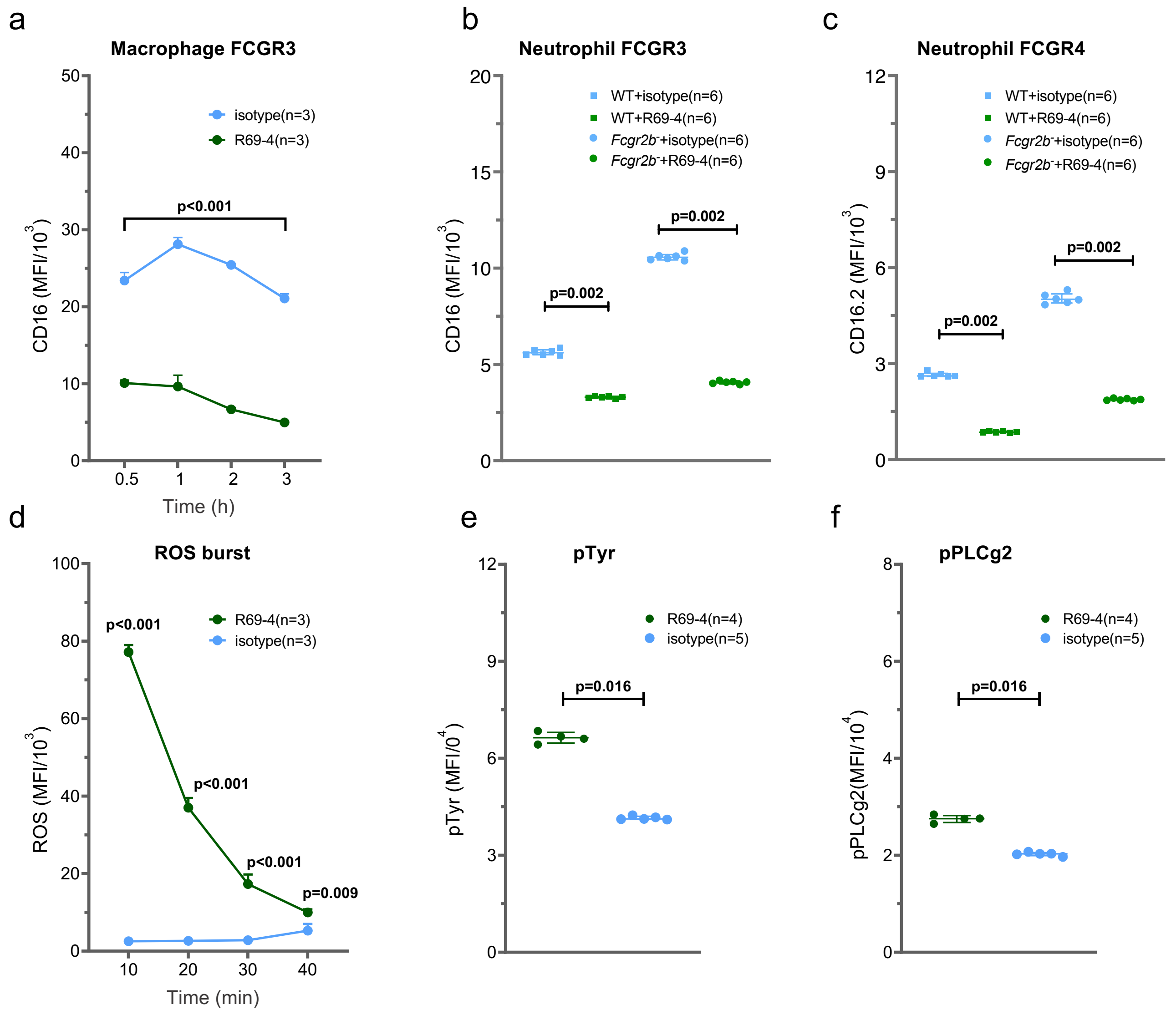

Fig S4. **a**. Bone marrow derived macrophages (BMDMs) incubated with R69-4 (100 µg/mL) expressed lower FCGR3 (Two-way ANOVA, $p<0.001$ from 0.5h to 3h). **b** and **c**. R69-4 (100 µg/mL) incubation induced down-regulation of both FCGR3 and FCGR4 on naïve BQ WT and *Fcgr2b*$^-$ neutrophils (Mann-Whitney U test, two-sided). **d**. Synovial fluid (SF) neutrophils collected from CAIA mice underwent rapid and robust reactive oxygen species (ROS) burst when incubated with R69-4 (100 µg/mL), whereas neutrophils cultured with isotype control did not (Two-way ANOVA). **e**. Intracellular staining illustrated an increase of phosphorylation of tyrosine containing motifs when SF neutrophils were incubated with R69-4 (100 µg/mL) (Mann-Whitney U test, two-sided). **f**. Intracellular staining illustrated that more phospholipase C gamma 2 (PLCγ2) was phosphorylated when SF neutrophils were incubated with R69-4 (100 µg/mL) (Mann-Whitney U test, two-sided). MFI: median fluorescent intensity.

Figure. S5

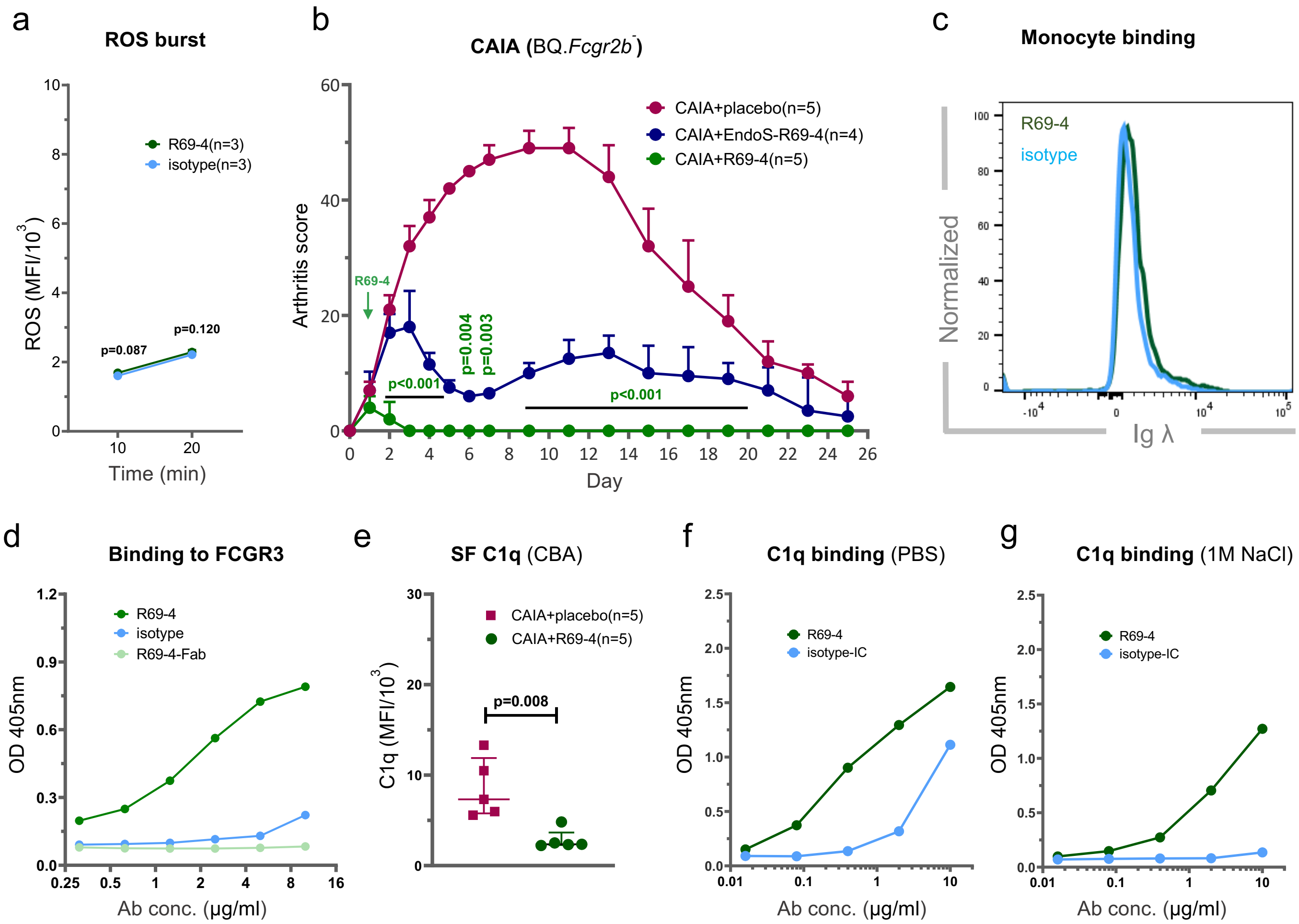

Fig S5. **a**. Synovial fluid (SF) neutrophils collected from CAIA mice treated with R69-4 failed to undergo intracellular ROS burst when incubated with R69-4 (100 µg/mL) or isotype control (Two-way ANOVA). **b.** Efficacy of Endo-S treated R69-4 in CAIA (BQ. *Fcgr2b*-). Cab4: 2 mg, d0, *i.v.*; R69-4 or Endo-S treated variant: 1 mg, d1, *i.v.*; Endo-S hydrolysis impaired the protective efficacy of R69-4 (Two-way ANOVA, p values indicate the post-hoc testing of R69-4 *vs* Endo-S-R69-4). **c**. No clear shift of Ig λ was observed for R69-4 compared to isotype control when incubated with monocytes; **d**. R69-4 bound to FCGR3 whereas R69-4-Fab did not. **e**. R69-4 treatment lowered the C1q levels in SF (Mann-Whitney U test, p=0.008). **f**. Both monomeric R69-4 and the control immune complex (IC, M2139-COL2) demonstrated binding to C1q in PBS; **g**. Monomeric R69-4 showed binding to C1q in 1M NaCl solution which prevents the binding between the Fc portion and C1q, whereas the control IC lost binding to C1q.

Figure. S6

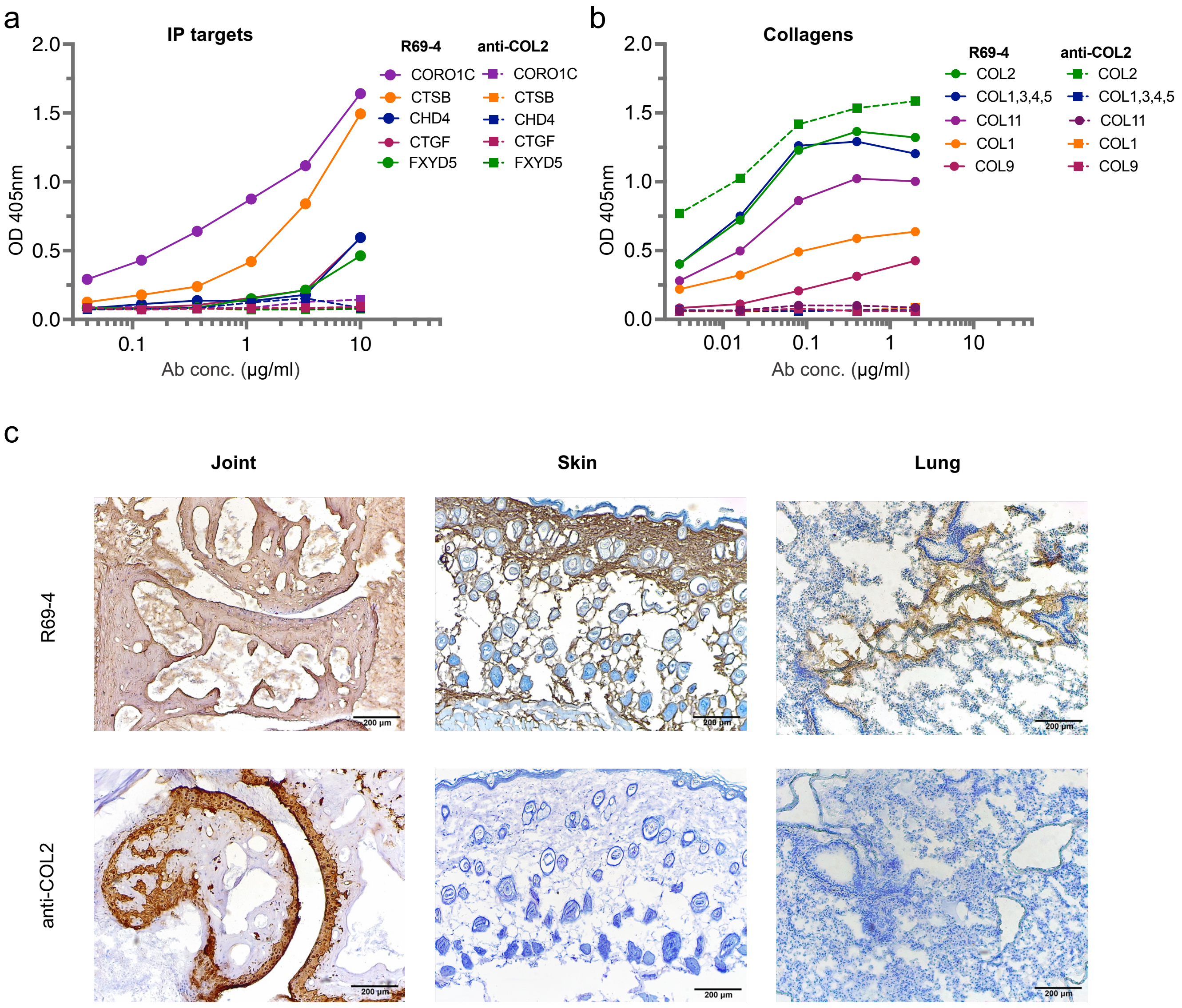

Fig S6. a. Binding of R69-4 or anti-COL2 control antibody (M2139) to available potential targets of R69-4 from immunoprecipitation (IP); **b**. Binding of R69-4 or anti-COL2 control antibody (M2139) to various collagen subtypes; **c**. Representative images showing that R69-4 not only bound to cartilage, but also to bone, lung, and skin tissues, whereas the anti-COL2 control antibody (M2139) stained cartilage exclusively. Positive staining is indicated by the reaction between horseradish peroxidase (HRP) and DAB substrate (brown). Scale bar: 200 µm.

Figure. S7 Gating strategy for flow cytometry data analysis

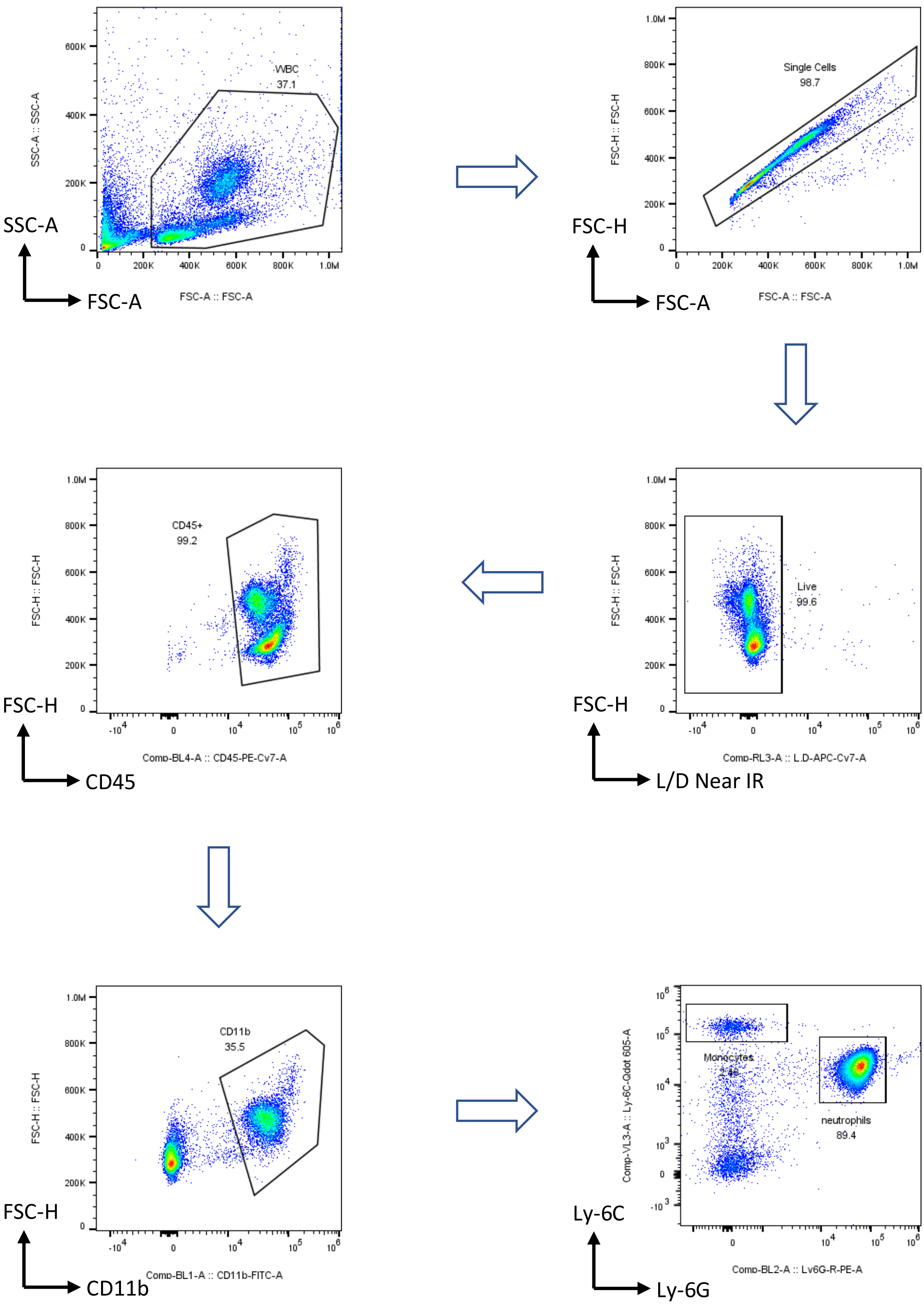

2. Supplementary Tables

Table S1. Representative ELISA and HTRF data for selected clones.

Clone	Selection track	re-ELISA (GFS-5) Abs 450 nm	re-ELISA (GFS-15) Abs 450 nm	re-ELISA (GFS-2) Abs 450 nm	re-ELISA (SA) Abs 450 nm	HTRF GFS-5	HTRF GFS-2	Native COL2	Denatured COL2
**scFv-1**	1:1:1:1_D10	3.54	3.57	0.01	0.02	1792	-7	0.19	-0.01
**scFv-2**	1:1:1:1_F08	3.74	3.73	0.18	0.16	2411	6	3.94	0.16
**scFv-3**	1:1:1:1_F10	2.57	3.13	0.01	-0.01	1316	-2	-0.01	-0.01
**scFv-4**	1:1:1:1_G10	3.76	3.64	0.25	0.18	2268	8	2.82	0.30
**scFv-5**	1:1:1:1_H08	3.81	3.77	0.20	0.06	708	10	0.89	0.00
**scFv-6**	1:2:1_A05	1.66	2.30	0.00	-0.01	488	21	-0.01	-0.02
**scFv-7**	1:2:1_B01	2.51	2.94	0.08	0.11	378	23	1.91	-0.01
**scFv-8**	1:2:1_C06	1.31	1.11	0.11	0.13	689	30	0.11	0.01
**scFv-9**	1:2:1_E05	2.16	1.60	0.06	0.08	838	25	-0.01	-0.02
**scFv-10**	1:2:1_G04	0.96	1.84	0.01	0.01	260	37	-0.01	-0.01
**scFv-11**	1:2:1:1_B11	3.11	3.39	0.04	0.12	455	13	0.00	-0.02
**scFv-12**	1:2:1:1_G07	3.39	3.01	0.03	0.04	1759	51	0.02	-0.02
**scFv-13**	1:2:1:1_G10	3.35	3.47	0.06	0.19	703	25	0.82	0.01
**scFv-14**	1:2:1:1_H07	3.64	3.63	0.05	0.12	2701	38	1.96	0.07
**scFv-15**	2:1:1_E03	0.58	0.51	0.05	0.05	214	22	-0.01	-0.02
**scFv-16**	2:1:1:1_B11	0.98	0.50	0.03	0.02	554	0	-0.01	-0.01
**scFv-17**	2:1:1:1_G11	1.70	0.98	0.08	0.09	500	26	2.21	-0.02
**scFv-18**	2:1:1:1_H10	3.64	3.84	0.09	0.21	2745	1	3.74	1.81
**scFv-19**	2:2:1_C02	3.70	3.73	0.11	0.03	1593	9	3.39	0.08
**scFv-20**	2:2:1_H01	3.24	3.24	0.23	0.25	813	12	2.69	0.05
**scFv-21**	2:2:1:1_A12	3.57	3.31	0.07	0.14	2013	-7	3.77	0.05
**scFv-22**	2:2:1:1_B07	3.66	3.78	0.43	0.19	1317	6	3.11	0.28
**scFv-23**	2:2:1:1_B10	3.51	3.63	0.07	0.12	554	-7	3.70	0.86
**scFv-24**	2:2:1:1_D08	3.61	3.73	0.14	0.17	807	2	2.26	0.04
**scFv-25**	2:2:1:1_E09	3.60	3.54	0.12	0.17	1102	-2	2.16	-0.01
**scFv-26**	2:2:1:1_E10	3.42	3.52	0.11	0.14	367	6	2.64	0.05
**scFv-27**	2:2:1:1_G07	1.48	1.13	0.07	0.04	1045	0	0.07	-0.01
**scFv-28**	2:2:1:1_G09	3.64	3.66	0.21	0.17	3324	26	0.47	0.04
**scFv-29**	2:2:1:1_H08	3.62	3.41	0.08	0.22	2203	0	3.45	0.01
**scFv-30**	3:1:1_A06	3.62	3.75	0.06	0.15	2284	4	2.02	0.00
**scFv-31**	3:1:1_B03	2.75	1.76	0.11	0.13	878	-3	0.06	-0.01
**scFv-32**	3:1:1_C01	3.48	3.44	0.02	0.03	1591	2	0.01	-0.01
**scFv-33**	3:1:1_C04	1.78	1.73	0.04	0.11	549	-6	0.03	-0.01
**scFv-34**	3:1:1_C05	2.97	3.03	0.04	0.08	997	9	0.50	0.00
**scFv-35**	3:1:1_D01	2.62	3.37	0.10	0.07	1043	32	0.02	-0.01
**scFv-36**	3:1:1_D06	3.36	3.48	0.00	0.00	1110	-2	-0.01	-0.02
**scFv-37**	3:1:1_G01	3.10	3.22	0.02	0.02	1526	16	0.25	0.00
**scFv-38**	3:1:1_H01	2.55	3.43	0.05	0.18	1112	1	0.00	-0.01
**scFv-39**	3:1:1_H02	1.30	1.60	0.03	0.09	698	-2	0.01	-0.01
**scFv-40**	3:1:1:1_C09	0.57	1.11	0.09	0.07	330	5	0.01	-0.01

**scFv-41**	3:1:1:1_D09	3.49	3.55	0.12	0.11	3091	-2	3.40	2.23
**scFv-42**	3:1:1:1_F09	1.67	2.56	0.04	0.09	1788	1	0.02	0.00
**scFv-43**	3:2:1_A01	3.26	3.61	0.01	0.02	823	-9	-0.01	-0.01
**scFv-44**	3:2:1_B03	0.81	1.42	0.03	0.01	452	-2	0.00	-0.01
**scFv-45**	3:2:1_C01	0.66	3.22	0.01	0.01	51	1	-0.01	-0.02
**scFv-46**	3:2:1_D06	3.50	3.69	0.18	0.18	3814	32	0.49	0.07
**scFv-47**	3:2:1_E01	3.63	3.67	0.43	0.05	1273	2	0.00	-0.01
**scFv-48**	3:2:1_G02	3.38	2.83	0.07	0.14	215	-7	0.08	0.01
**scFv-49**	3:2:1_G03	3.44	3.46	0.05	0.11	1653	6	0.05	0.00
**scFv-50**	3:2:1:1_B12	3.48	3.61	0.01	0.00	1018	1	0.10	0.00
**scFv-51**	3:2:1:1_C07	2.65	3.42	0.02	0.04	426	6	0.00	0.00
**scFv-52**	3:2:1:1_F10	3.51	3.66	0.03	0.02	2057	-6	0.00	-0.01
**scFv-53**	3:2:1:1_H10	3.56	3.38	0.05	0.09	1679	-5	3.94	0.00
**scFv-54**	4:1:1_B04	3.50	3.52	0.05	0.11	774	41	0.11	0.01
**scFv-55**	4:1:1_C01	2.87	3.41	-0.01	0.00	825	-1	-0.01	-0.02
**scFv-56**	4:1:1:1_B11	3.64	3.69	0.09	0.11	765	1	0.49	0.07
**scFv-57**	4:1:1:1_C08	3.41	3.49	0.14	0.13	1001	2	3.30	0.01
**scFv-58**	4:1:1:1_C10	0.88	2.25	0.07	0.06	323	21	0.00	-0.01
**scFv-59**	4:1:1:1_G08	1.30	1.84	0.06	0.06	419	13	0.03	-0.01
**scFv-60**	4:1:1:1_G09	3.94	3.94	0.16	0.16	2797	35	3.64	0.52
**scFv-61**	4:2:1_A01	0.63	1.03	0.15	0.07	284	-2	0.10	0.00
**scFv-62**	4:2:1_B06	0.79	1.77	0.09	0.08	724	-3	0.00	-0.01
**scFv-63**	4:2:1_C03	0.24	0.33	0.00	0.00	62	11	0.00	-0.01
**scFv-64**	4:2:1_C04	1.57	2.12	0.10	0.16	1203	-1	0.02	0.00
**scFv-65**	4:2:1_C05	0.44	1.07	0.05	0.03	201	-4	0.00	-0.01
**scFv-66**	4:2:1_C06	1.04	2.01	0.00	0.00	784	-1	-0.01	-0.01
**scFv-67**	4:2:1_D04	2.12	3.67	0.08	0.05	584	-5	0.04	-0.01
**scFv-68**	4:2:1_D06	2.55	3.17	0.05	0.07	265	-4	0.01	-0.01
**scFv-69**	4:2:1_E06	3.74	3.78	0.08	0.01	1905	7	1.20	1.98
**scFv-70**	4:2:1_H05	3.66	3.68	0.15	0.05	378	7	0.00	0.00
**scFv-71**	4:2:1:1_A07	3.57	3.51	0.00	0.01	2324	-7	-0.02	-0.01
**scFv-72**	4:2:1:1_A09	2.97	3.26	0.10	0.09	1829	-8	0.02	0.00
**scFv-73**	4:2:1:1_C10	3.36	3.26	0.08	0.06	1440	-15	0.01	-0.01
**scFv-74**	4:2:1:1_E07	2.81	3.51	0.05	0.06	407	-3	2.99	0.23
**scFv-75**	4:2:1:1_F07	3.56	3.49	0.05	0.05	1733	5	2.60	0.23
**scFv-76**	4:2:1:1_F08	3.79	3.56	0.05	0.03	860	5	0.05	-0.01
**scFv-77**	4:2:1:1_G07	0.80	1.66	0.07	0.08	206	4	0.00	-0.01
**G-Strep-1**		0.44	0.63	0.35	2.61	3315	3167	0.01	-0.01
**Blank**		-0.01	-0.01	0.00	0.00	1	-7	-0.01	-0.01
**Anti-F4**		3.19	3.19	0.04	0.07	-5	2	1.69	0.18

Table S2. Summary of selected clones on their binding to relevant targets.

Clone	Cartilage	SynF4-24aa	RecF4-24aa	SynF4-12aa
scFv-4	Strong	+	+	-
scFv-18	Strong	+	+	+
scFv-2	Mild	+	+	-
scFv-7	Mild	+	-	+
scFv-25	Mild	+	-	-
scFv-28	Mild	+	+	+
scFv-29	Mild	+	-	-
scFv-30	Mild	+	-	-
scFv-34	Mild	+	+	-
scFv-41	Mild	+	+	-
scFv-53	Mild	+	+	-
scFv-57	Mild	+	-	-
scFv-60	Mild	+	+	+
scFv-14	Weak	+	+	+
scFv-23	Weak	+	-	+
scFv-24	Weak	+	-	-
scFv-26	Weak	+	-	-
scFv-19	-	+	+	+
scFv-21	-	+	+	-
scFv-36	-	+	+	-
scFv-46	-	+	+	-
scFv-47	-	+	-	+
scFv-49	-	+	+	-
scFv-50	-	+	+	-
scFv-69	-	+	-	-
scFv-70	-	-	-	-

Cartilage: binding to neonatal cartilage tissue determined by immunohistochemistry (IHC). SynF4-24aa: synthesized F4 peptide containing 24 amino acids; RecF4-24aa: recombinant F4 peptide containing 24 amino acids; SynF4-12aa: synthesized F4 peptide containing 12 amino acids. The binding to peptides was determined by bead-based flow immunoassay, and all peptides are triple helix.

Table S3. Potential R69-4 binding targets screened by immunoprecipitation (IP) and ELISA.

Protein name	p value (R69-4)	FDR (R69-4)	Fold change (R69-4)	p value (isotype)	FDR (isotype)	Fold change (isotype)	Validation
**Ctgf**	0.011	0.044	4.19	0.962	0.975	1.02	validated
**Npc1**	0.016	0.054	3.50	0.398	0.528	0.60	-
**Fcgr3**	0.048	0.104	3.31	0.518	0.629	0.70	Fc interaction
**Gpd1l**	0.033	0.082	3.14	0.627	0.722	1.13	-
**Chd4**	0.041	0.096	3.05	0.373	0.503	0.67	validated
**Pef1**	0.036	0.087	3.02	0.775	0.836	1.09	-
**Tapbp**	0.008	0.037	2.64	0.838	0.879	1.13	-
**Tbc1d10b**	0.043	0.098	2.57	0.941	0.959	0.96	-
**Fxyd5**	0.035	0.086	2.45	0.307	0.443	0.61	validated
**Cct4**	0.043	0.098	2.34	0.694	0.774	1.11	-
**Cnbp**	0.0001	0.004	2.21	0.662	0.750	0.91	-
**Hnrnpab**	0.022	0.066	2.04	0.771	0.832	1.08	-
**Marcks**	0.012	0.046	2.03	0.838	0.880	1.07	-
**Coro1c**	0.002	0.020	1.99	0.458	0.580	1.14	validated
**Igfbp4**	0.041	0.096	1.92	0.636	0.729	1.17	-
**Rps26**	0.015	0.053	1.77	0.064	0.248	0.65	-
**Aldh3b1**	0.016	0.055	1.74	0.728	0.800	1.10	-
**Arsb**	0.028	0.075	1.73	0.541	0.648	1.19	-
**Ppid**	0.036	0.087	1.64	0.551	0.656	1.15	-
**Ctsb**	0.040	0.093	1.59	0.361	0.494	1.17	validated
**Sap18**	0.043	0.098	1.58	0.985	0.991	1.01	-
**Ncf2**	0.009	0.041	1.48	0.617	0.715	1.12	-
**C1q**	-						ELISA
**COL1**	-						ELISA
**COL2**	-						ELISA
**COL9**	-						ELISA
**COL11**	-						ELISA

P values and FDR calculated by using Student's t test followed by Storey-Tibshirani procedure for the correction of multiple hypothesis testing. Validation was performed using ELISA.

3. Supplementary notes: Screening of recombinant antibodies

3.1 Materials for screening

Reagents

Selection antigens (triple-helical peptides, in house production)
Aliquots of the phage display antibody library (SciLife Lab)
Phosphate buffered saline (P4417-100TAB, Sigma-Aldrich)
PBS/Tween 20 (0.1%)
Skimmed milk powder (Marvel)
2xTY medium (Y2377-250G, Sigma-Aldrich)
2xTYAG media (2xTY plus 100μg/mL Ampicillin and 2% (w/v) glucose)
Trypsin (T8003, Sigma-Aldrich)
0.1M Sodium phosphate buffer (pH 7.0)
Fresh minimal media agar plates with TG1 cells
FastDigest *Kpn*I restriction enzyme (FD0524, Thermo Scientific)
FastDigest *Hind*III restriction enzyme (FD0504, Thermo Scientific)
FastDigest *Nhe*I restriction enzyme (FD0974, Thermo Scientific)
T4 DNA ligase (EL0011, Thermo Scientific)
GeneJET plasmid mini prep kit (K0503, Thermo Scientific)
GeneJET Gel extraction kit (K0692, Thermo Scientific)
GeneJET PCR purification kit (K0702, Thermo Scientific)
Plasmid DNA mega kit (12181, Qiagen)
UltraPureAgarose (16500-500, Invitrogen by Life technologies)
DH5α chemical competent cells (EC0112, ThermoFisher Scientific)
LB Broth (Lennox) (L3022, Sigma-Aldrich)
Expi293 Expression Medium (A14351-01, Gibco by Life technologies)
Opti-MEM (1x) Reduced Serum Medium (31985-070, Gibco by Life technologies)
FectoPRO transfection reagent (116-010, Polyplus)
5 mL HiTrap ProteinG (Cytiva)
NuPAGE 4-12% Bis-Tris Gel (NP0323BOX, Invitrogen by Life technologies)
DELFIA Eu-labeled N1 rabbit anti-mouse IgG antibody (AD0124, Delfia)
HRP Anti-6X His tag® antibody (ab1187, Abcam)
ABTS tablets (11204521001, Roche Diagnostic GambH)
Monoclonal Anti-FLAG® M2 antibody produced in mouse (F3165, Sigma-Aldrich)
Rat Type II collagen (Batch #61, In house production)
4% Formaldehyde phosphate buffered (Histolab)
Extra Avidin® peroxidase (E2886 – 1 mL, Sigma)
DAB peroxidase substrate kit (SK-4100, Vector Laboratories, Inc Burlingame, CA94010)

Phage display library

Two human phage display libraries, designated SciLifeLib1 and SciLifeLib2, were used for selection. The libraries are constructed scFv consisting of two variable immunoglobulin domains with one derived from the heavy chain and one from the light chain. The light chain and heavy chain are covalently linked by a $(Gly_4\text{-}Ser)_3$ linker. The four framework sequences in the two libraries are constant whereas the three complementarity determining regions (CDRs) are different. In addition, FLAG and 6xHis tags were added at the N-terminal of the sequence. The diversity of the libraries is up to $10e^9$ approximately.

Synthesized peptides for phage display selection

Peptides used for selection were GFS-5, GFS-15, and GFS-2. The GFS-5 peptide is shown in **Fig. Sn1**. The collagen peptide with 24 amino acids containing the F4 epitope is flanked by 5 GPO repeats on both N- and C- terminal. At the C-terminal of the 5 GPO, a lysine knot is added to covalently link the peptide to form a stable triple helical structure. The 3 peptides were biotinylated at the C-terminal. The GFS-15 peptide has the same arrangement as GFS-5 except the first arginine residue that is replaced by citrulline. GFS-2 is the C1 epitope containing triple helical peptide used as counter screen.

Fig Sn1. Sequence structure of the GFS-5 triple helix peptide.

(GPO)5- GDKGEAGEOGERGLKGHRGFTGLQ - (GPO)5-Ahx-KKGYG-CONH2-biotin
(GPO)5- GDKGEAGEOGERGLKGHRGFTGLQ - (GPO)5-Ahx
(GPO)5- GDKGEAGEOGERGLKGHRGFTGLQ - (GPO)5-Ahx

3.2 Phage display selection and screening

Phage display selection

Summary of selection procedure:

- Coat Streptavidin Dynabeads with antigen (biotinylated peptides)
- Inoculate TG1 culture
- Block biotinylated peptides coated Streptavidin Dynabeads
- Pre-block phage
- Incubate phage library with coated antigen
- Wash to remove unbound phage
- Elute bound phage with trypsin
- Infect TG1 with eluted phage with second-round selection

Three biotinylated peptides, GFS-5, GFS-15, and GFS-2, respectively, were mixed with Streptavidin Dynabeads. The coupling was allowed overnight at 4°C. After rinsing 3 times with PBS to remove unbound peptides, the Dynabeads were blocked with 3%

(w/v) skimmed milk powder in PBS and incubated for 1 hour at ambient temperature. The coated Dynabeads were rinsed again with PBS and the pre-blocked phage was added and incubated at 37°C for 1 hour. Unbound phages were removed by washing 5 times with PBS/0.1% Tween followed by 5 times with PBS. Bound phages were eluted with trypsin at 10 µg/mL in 0.1 M sodium phosphate buffer (pH7.0) for 30 min. The eluted phages were then added to a mid-log culture of *E.coli* TG1 cells for enrichment. The culture was incubated for 1 hour at 37°C and 150 rpm. The enriched phage display libraries were used for a second selection round. In total four selection rounds were performed and the concentration of the peptides in each round was reduced to increase the stringency. The selection strategy and the peptide concentrations are shown in **Table. Sn1**.

Table. Sn1. Selection strategy for GFS-5, GFS-15, and GFS-2 peptides with two phage display libraries

**Selection**	**SciLifeLib1**<br>**Target = GFS-5**		**SciLifeLib2**<br>**Target = GFS-5**	
**R1**	**Track**<br>500 nM GFS-2<br>250 nM GFS-5<br>Incubation 3 h<br>Wash 5 x 0.8 ml PBST		**Track 2**<br>500 nM GFS-2<br>250 nM GFS-5<br>Incubation 3 h<br>Wash 5 x 0.8 ml PBST	
**R2**	**Track 1:1**<br>500 nMGFS-2<br>100 nM GFS-5<br>Incubation 1 h<br>Wash 5 x 0.8 ml PBST	**Track 1:2**<br>500 nM GFS-2<br>100 nM GFS-15<br>Incubation 1 h<br>Wash 5 x 0.8 ml PBST	**Track 2:1**<br>500 nMGFS-2<br>100 nM GFS-5<br>Incubation 1 h<br>Wash 5 x 0.8 ml PBST	**Track 2:2**<br>500 nM GFS-2<br>100 nM GFS-15<br>Incubation 1 h<br>Wash 5 x 0.8 ml PBST
**R3**	**Track 1:1:1**<br>100 nM GFS-2<br>25 nM GFS-5<br>Incubation 1 h<br>Wash 6 x 0.8 ml PBST	**Track 1:2:1**<br>100 nM GFS-2<br>25 nM GFS-5<br>Incubation 1 h<br>Wash 6 x 0.8 ml PBST	**Track 2:1:1**<br>100 nM GFS-2<br>25 nM GFS-5<br>Incubation 1 h<br>Wash 6 x 0.8 ml PBST	**Track 2:2:1**<br>100 nM GFS-2<br>25 nM GFS-5<br>Incubation 1 h<br>Wash 6 x 0.8 ml PBST
**R4**	**Track 1:1:1:1**<br>20 nM GFS-2<br>5 nM GFS-5<br>Incubation 1 h<br>Wash 9 x 0.8 ml PBST	**Track 1:2:1:1**<br>20 nM GFS-2<br>5 nM GFS-15<br>Incubation 1 h<br>Wash 9 x 0.8 ml PBST	**Track 2:1:1:1**<br>20 nM GFS-2<br>5 nM GFS-5<br>Incubation 1 h<br>Wash 9 x 0.8 ml PBST	**Track 2:2:1:1**<br>20 nM GFS-2<br>5 nM GFS-15<br>Incubation 1 h<br>Wash 9 x 0.8 ml PBST

**Selection**	**SciLifeLib1**<br>**Target = GFS-15**		**SciLifeLib2**<br>**Target = GFS-15**	
**R1**	**Track 3**<br>500 nM GFS-2<br>250 nM GFS-15<br>Incubation 3 h<br>Wash 5 x 0.8 ml PBST		**Track 4**<br>500 nM GFS-2<br>250 nM GFS-15<br>Incubation 3 h<br>Wash with King Fisher 5 x 0.8 ml PBST	
**R2**	**Track 3:1**<br>500 nM GFS-2<br>100 nM GFS-15<br>Incubation 1 h<br>Wash 5 x 0.8 ml PBST	**Track 3:2**<br>100 nM GFS-5/SA<br>100 nM GFS-15<br>Incubation 1 h<br>Wash 5 x 0.8 ml PBST	**Track 4:1**<br>500 nM GFS-2<br>100 nM GFS-15<br>Incubation 1 h<br>Wash 5 x 0.8 ml PBST	**Track 4:2**<br>100 nM GFS-5/SA<br>100 nM GFS-15<br>Incubation 1 h<br>Wash 5 x 0.8 ml PBST
**R3**	**Track 3:1:1**<br>100 nM GFS-2<br>25 nM GFS-15<br>Incubation 1 h<br>Wash 6 x 0.8 ml PBST	**Track 3:2:1**<br>100 nM GFS-5<br>25 nM GFS-15<br>Incubation 1 h<br>Wash 6 x 1 ml PBST	**Track 4:1:1**<br>100 nM GFS-2<br>25 nM GFS-15<br>Incubation 1 h<br>Wash 6 x 0.8 ml PBST	**Track 4:2:1**<br>100 nM GFS-5<br>25 nM GFS-15<br>Incubation 1 h<br>Wash 6 x 0.8 ml PBST
**R4**	**Track 3:1:1:1**<br>20 nM GFS-2<br>5 nM GFS-15<br>Incubation 1 h<br>Wash 9 x 0.8 ml PBST	**Track 3:2:1:1**<br>20 nM GFS-5<br>5 nM GFS-15<br>Incubation 1 h<br>Wash 9 x 0.8 ml PBST	**Track 4:1:1:1**<br>20 nM GFS-2<br>5 nM GFS-15<br>Incubation 1 h<br>Wash 9 x 0.8 ml PBST	**Track 4:2:1:1**<br>20 nM GFS-5<br>5 nM GFS-15<br>Incubation 1 h<br>Wash 9 x 0.8 ml PBST

**Selection**	**SciLifeLib1**<br>**Target = GFS-2**	**SciLifeLib2**<br>**Target = GFS-2**
**R1**	**Track 5**<br>500 nM GFS-5/15-<br>300 nM GFS-2<br>Incubation 1 h<br>Wash 5 x 0.8 ml PBST	**Track 6**<br>500 nM GFS-2/15-<br>300 nM GFS-2<br>Incubation 1 h<br>Wash 5 x 0.8 ml PBST
**R2**	**Track 5:1**<br>300 nM GFS-5/15-<br>75 nM GFS-2<br>Incubation 1 h<br>Wash 6 x 0.8 ml PBST	**Track 6:1**<br>300 nM GFS-5/15-<br>75 nM GFS-2<br>Incubation 1 h<br>Wash 6 x 0.8 ml PBST
**R3**	**Track 5:1:1**<br>100 nM GFS-5/15-<br>15 nM GFS-2<br>Incubation 1 h<br>Wash 9 x 0.8 ml PBST	**Track 6:1:1**<br>100 nM GFS-5/15-<br>15 nM GFS-2<br>Incubation 1 h<br>Wash 9 x 0.8 ml PBST

Primary screening

ELISA

To the Streptavidin plate, biotinylated peptides GFS-5, GFS-15 and GFS-2 were added at a concentration of 2 µg/mL, respectively. After incubation at 37°C for 1 hour, the scFv containing bacteria culture supernatant was added and incubated at 37°C for 1 hour. The detection antibody used was the anti-FLAG-HRP conjugate (Sigma-Aldrich) and the plates were incubated at 37°C for 1 hour followed by washing and addition of the ABTS substrate. The plates were read at 405 nm.

Homogeneous Time Resolved Fluorescence (HTRF)

FRET (Fluorescence Resonance Energy Transfer) is based on the transfer of energy between two fluorophores, a donor, and an acceptor, when in close proximity. Molecular interactions between biomolecules can be assessed by coupling each partner with a fluorescent label and detecting the level of energy transfer. When two entities come close enough to each other, excitation of the donor by an energy source triggers an energy transfer towards the acceptor, which in turn emits specific fluorescence at a given wavelength. The donor and acceptor can be grafted covalently onto multiple partners that can associate for an antigen and an antibody. To the GFS-5 coated beads, scFv was added followed by mixing the Ms-anti-FLAG-Tb (320 nm) antibody (Cisbio #611FG2TL) and Streptavidin-XL665 665 nm) (Cisbio #610SAXL).

DNA sequencing (GATC Biotech, Germany)

After primary screen by ELISA, positive clones were sent for sequence analysis at GATC Biotech (Germany).

Secondary screen of unique clones

Secondary screen was done on unique clones by ELISA for their binding to native and denatured type II collagen.

For denatured type II collagen ELISA, the type II collagen was diluted in PBS to a final concentration of 10 µg/mL, heated at 60°C for 30 min followed by immediate coating and incubation of the plate at 37°C for 40 min.

For native type II collagen ELISA, the collagen was diluted in PBS to final concentration of 10 µg/mL and 100 µL/well was added to the plate.

Then the phage display culture supernatant was added and incubated at 37°C for one hour. After washing, anti-FLAG-HRP was added, and the plates were incubated at 37°C for 1 hour followed by washing and addition of the ABTS substrate. The plates were read at 405 nm.

3.3 Production of scFv for further screen

SciLife Lab: scFv in *E.coli* TOP10 was grown in LB media containing 50 mg/mL Carbenicillin. After overnight culture, the bacteria were pelleted and lysed through periplasmic extraction. The supernatant was then mixed with Protein A magnetic beads (Thermo Scientific #88845) using the Kingfisher instrument. After washing the beads, the target protein was eluted at low pH (100 mM glycin, pH 2.7) diafiltrated and stored in 1 x PBS at 4°C. The purified scFv was analyzed by SDS-PAGE under reducing conditions and the protein concentration was determined using the BCA method using BSA as standard protein.

Kinetic screen using Biacore

The interaction of scFv's with the native type II collagen was assessed by SPR using a Biacore T200 biosensor (GE Healthcare) at 25°C. Anti-FLAG antibody (Sigma-Aldrich #F1840) was coupled covalently to the carboxymethylated dextran of a CM5 sensor chip through amine coupling according to the manufacturer's instruction. The scFv was injected into the chip at 10 µL/min and captured through anti-FLAG/FLAG tag interaction. The collagen (10 µg/mL) was then injected over the sensor chip surface for 60 seconds at a flow rate of 30 µL/min, followed by a 300 second dissociation phase. A regeneration solution (glycine-HCl, pH 1.7) was used before each run to remove all previously bound molecules. The thermodynamic (KD) and kinetic (Kon and Koff) parameters of the scFv binding COL2 were determined by single-cycle kinetics. Sensorgrams were processed using an automatic correction for nonspecific bulk-refractive-index effects. Data processing and analysis were performed using Biacore T200 evaluation software in a 1:1 binding model (GE Healthcare).

Luminex analysis

An in-house peptide library was created for various purposes. The peptide library contains: 1) over-lapping peptides covering the entire type II collagen sequence; 2) Arginine residue-containing peptides from other proteins, such as GPI, COMP and PAD4; 3) Arginine residue-containing cyclic peptides from several proteins. In addition, the same set of peptides but with citrulline instead of arginine were also synthesized. The selected unique clones were screened on the entire peptide library using the Luminex technique. For comparison, both FLAG (R-PE) and His-tagged (R-PE) secondary antibodies were tested.

Histology staining

Described in the main text.

3.4 Conversion to full length antibodies

Construct design

In-house, we have already created a vector that contains a mouse IgG2b constant region with KpnI/HindIII restriction sites (*Kpn*I at the 5' end and *Hind*III at the 3' end of the coding sequence) for the insertion of heavy chain variable region and another vector containing a mouse lambda constant region with KpnI/NheI sites for the insertion of light chain variable region. The 2 vectors were designated with pCEP4-mIgG2b and pCEP4-mL. After identification of the variable heavy chain and light chain sequences from the scFv sequence, respectively, the variable region of the heavy chain and light chain sequences were synthesized with incorporated respective restriction sites. For secretion of the protein into the culture media, a signal sequence was included.

Restriction enzyme digestion and agarose gel electrophoresis

All genes were synthesized at Eurofins Medigenomix GmbH (Ebersberg, Germany) in the standard cloning vector pEX-A2.
The synthesized gene was diluted with water to a final plasmid concentration of 100 ng/µL. The restriction enzyme digestion mix contained: 1) 2 µL 10x FD buffer, 1.5 µL *Kpn*I, 1.5 µL *Hind*III and 15 µL synthesized variable HC gene; and 2) 2 µL 10x FD buffer,

1.5 µL *Kpn*I, 1.5 µL *Nhe*I and 15 µL synthesized variable LC gene. After incubation at 37 °C for 15 min, the entire mix was loaded onto a 1% agarose gel together with a 1 kb marker. Products around 450 bp were cut from the agarose gel and DNA was extracted using the GeneJET Gel extraction kit (K0692). Meanwhile the expression vector pCEP4-mIgG2b and pCEP4-mL were also digested using the respective restriction enzymes and purified using the GeneJET PCR purification kit (K0702). The DNA was eluted in 30 µL elution buffer.

Ligation and transformation

The purified heavy and light chain variable region-coding sequences were directionally cloned into the pCEP4-mIgG2b and pCEP4-mL vectors. 2 µL 10xT4 ligation buffer, 1 µL (25 ng) digested pCEP4 vector, 2 µL insert, 15 µL H_2O and 0.4 µL T4 DNA ligase were mixed in a total volume of 20 µL. The ligation mix was incubated at ambient temperature (around 20°C) for 30 min. 4 µL ligation mix was added to 50 µL DH5α chemical competent cells for the transformation. The cells were put on ice for 5 min and subjected to heat shock at 42 °C for 40 s. The cells were then placed on ice for another 2 min. Five hundred µL SOC media (Invitrogen) was added and the *E.coli* were placed on a shaker (MaxQ 4000, Thermo Scientific) at 37 °C, 200 rpm for 1 hour. The *E.coli* transformants were then selected on agar plates containing the antibiotic Carbenicillin (Invitrogen) at a concentration of 50 µg/mL. The plates were incubated at 37°C overnight.

Colony screen using restriction enzyme digestion followed by sequencing analysis

Four colonies from each transformation were picked from the LA plates and inoculated into 3 mL LB media containing 50 µg/mL Carbenicillin, respectively, followed by shaking at 200 rpm at 37°C overnight. The plasmid DNAs were prepared using the "GeneJET Plasmid miniprep Kit (K0503)" according to the manufacturer's instruction. 50µL TE buffer was added to elute the plasmid DNA. Confirmation of the right insert size was done by restriction enzyme digestion. The reaction mix contained 0.2 µL *Kpn*I, 0.2µL *Bam*HI, 1.0 µL 10 x FD Buffer, 7.6 µL H_2O and 1.0 µL plasmid. The mix was incubated at 37˚C for 15 min. The digests were analyzed by 1.0% agarose gel electrophoresis. All inserts with the right size at around 1500 bp for the heavy chain and 900 bp for the light chain were sequenced using the BigDye Terminatorv3.1 Cycle Sequencing Kit (Thermo Scientific). The plasmids with right sequences were used for small-scale expression. To this end, for each antibody two plasmids, one containing the heavy chain and one containing the light chain were needed to co-transfect into the mammalian cells.

Plasmid DNA mega prep

When the small-scale experiments showed expression of the functional antibodies, more plasmids were prepared for transient transfection using the QIAGEN Plasmid Mega Kit (12183, Qiagen) according to manufacturer's instruction.

30 mL testing of expression

The day before transfection, 27 mL of the Expi293F^{TM} (Life technologies) cell suspension at 1 x 10^6 cells/mL was prepared and the cells were incubated overnight on an incubation shaker (Minitron, Infors HT) at 37°C, 120 rpm and 8% CO_2. On the day of transfection, 24 µL transfection reagent, FectoPRO (Polyplus transfection), was added to a 15 mL Falcon tube containing 1.5 mL Opti-MEM (1x) Reduced Serum Medium (Gibco) followed by gentle vortex. In a second tube, 9.6 µg heavy chain-containing plasmid and 14.4 µg light chain-containing plasmid were added to 1.5 mL Opti-MEM (1x) Reduced Serum Medium. After gentle vortex, the diluted DNA was immediately mixed with the diluted FectoPRO transfection reagent and incubated for 10 minutes at room temperature followed by the addition of 3 mL FectoPRO/DNA transfection mix to the cells with gentle stirring. The cells were incubated at 37°C, 120 rpm and 8% CO_2 and the supernatant was harvested around 6 days post-transfection or when viability dropped to around 50%. The supernatant was analyzed by SDS-PAGE and ELISA.

Lab scale production

For lab scale production, the same procedure was used as for the 30 mL scale expression. The process volume was scaled up proportionally to 500 mL. The day before transfection, 450 mL of the Expi393F^{TM} (Life technologies) cell suspension at 1 x 10^6 cells/mL was prepared and the cells were incubated overnight at 37°C, 120 rpm and 8% CO_2. On the day of transfection, 400 µL transfection reagent, FectoPRO (Polyplus transfection) was added to a 50 mL Falcon tube containing 20 mL Opti-MEM (1x) Reduced Serum Medium (Gibco) with gentle vortex. In a second tube, 160 µg heavy chain-containing plasmid and 240 µg light chain-containing plasmid was added to 20 mL Opti-MEM (1x) Reduced Serum Medium. After gentle vortex, the diluted DNA was mixed with the diluted FectoPRO transfection reagent followed by immediate vortex. After incubation for 10 minutes at room temperature, the 40 mL FectoPRO/DNA transfection mix was added to the cells with gentle shaking. The cells were incubated at 37°C, 120 rpm and 8% CO_2 and the supernatant was harvested around 6 days post-transfection or when viability dropped to around 50%. Around 500 mL supernatant was harvested after production for 6 days. After sterile filtration, the supernatant was stored at 4˚C before purification.

Affinity chromatography using a HiTrap Protein G HP column

A 5 mL HiTrap Protein G HP column (GE Healthcare Life Sciences) was equilibrated in PBS at a flow rate of 2.0 mL/min for 5 CVs. The supernatant was loaded at the same flow rate. After sample application, unbound material was washed away with equilibrium buffer for 5 CVs. The bound proteins were eluted using a 100 mM Glycine-HCl buffer, pH 2.7. The whole peak was collected.

Dialysis and concentration

The eluate from the affinity chromatography column was dialyzed against 10 mM glycine containing 150 mM NaCl to avoid aggregates. The dialyzed antibody solution was then concentrated to between 10-15 mg/mL using an Amicon Centriplus ultrafiltration membrane with a molecular weight cut-off of 30kDa.

3.5 Analysis of full-length antibodies

Protein concentration determination

The protein concentration was measured at 280 nm using the Nanodrop (Thermo Scientific) instrument, and the concentration was calculated by dividing the A280 nm with the extinction coefficient value.

SDS-PAGE

The culture supernatant and the purified antibodies were analyzed by SDS-PAGE under both reducing and non-reducing conditions. NuPAGE^{TM} 4-12% Bis-Tris pre-cast Gel (NP0341BOX) sourced from Invitrogen was used. The experiment was performed following the manufacturer's instruction.

ELISA on native collagen

1. Native type II collagen was diluted in PBS to 5 µg/mL and 100 µL was added to a 96-well plate (MaxSorp, Nunc). The plate was incubated at 4°C overnight.
2. The culture supernatant and the purified proteins were diluted in 5-fold series with blocking buffer (PBS/0.05% Tween-20 containing 1% BSA) and added to the plate followed by incubation at 37˚C for 1 hour.
3. The plate was washed four times and 100 µL of 1:1000 diluted Europium-labeled N1 rabbit anti-mouse-IgG antibody (AD0124, Delfia) was added to each well followed by incubation at 37˚C for 1 hour.
4. The plate was washed four times and 100 µL of Delfia enhancement solution were added.
5. The fluorescence was read after 5 min of incubation at ambient temperature.

ELISA on peptides

1. GFS-5, GFS-15 and mutated F4 peptides were diluted in PBS to 5 µg/mL and 100 µL were added to a 96 well plate (MaxSorp, Nunc). The plates were incubated at 4°C overnight.
2. The culture supernatant and the purified proteins were diluted in 5-fold series with blocking buffer (PBS/0.05% Tween-20 containing 1% BSA) and added to the plate followed by incubation at 37˚C for 1 hour.
3. The plate was washed four times and 100 µL of 1:1000 diluted Europium-labeled N1 rabbit anti-mouse-IgG antibody (AD0124, Delfia) was added to each well followed by incubation at 37˚C for 1 hour.
4. The plate was washed 4 times and 100 µL of Delfia enhancement solution were added.
5. The fluorescence was read after 5 min incubation at ambient temperature.

Luminex study

The same procedure was used as described previously.

3.6 Cartilage Antibody Induced Arthritis (CAIA)

BQ.Cia9i mice aged 3 to 4 months were used in the CAIA experiment. In total, there were 6 groups with 5 mice per group. On day 0, all mice received 4.5 mg M2139 and 4.5 mg CIIC1 by intravenous injection and on day 1, R69-4, 7, 14, 18 and 19 antibodies were given, respectively to 5 groups with 4.5 mg/mouse. In addition, one group received PBS as control. On day 4, all mice were boosted with a high dose (50 µg) of lipopolysaccharide (LPS) by intra-peritoneal injection, due to the low potency of antibody cocktail containing two antibodies. Arthritis severity was scored every second day until 3 weeks post cocktail injection.

3.7 Screening results

Peptide selection

Several versions of peptides have been synthesized for various applications. For epitope mapping, longer peptides (with both triple helical structures and linear structures) with 24 amino acid residues were synthesized, whereas short peptides with 12 amino acid residues were mainly used for crystallization purposes. In addition, cyclic peptides have been synthesized for diagnostic applications. The longer peptides were used for phage display selection because of their higher stability. The short peptides (12 aa) are less stable as analyzed by thermo-shift with lower melting temperature (20°C). The longer peptides have a thermo-shift at around 40°C and the selection took place at 37°C. Also, CD spectra showed the typical triple-helical structure for both short and long peptides, but with less triple-helix content in comparison with the longer peptides.

Magnetic Streptavidin beads were used to couple the synthesized biotinylated peptides. To make the selection peptides perform as natively as possible, triple helix structures were needed. For holding the triple helix structure, 5 repeated GPO sequences were included at both N- and C-terminal of the peptides. To avoid selection based on the GPO parts of the sequence, a counter selection was used with peptides that also contains 5 GPO repeats but with another collagen C1 epitope (GFS-2).

Phage display selection and primary screen

SciLife Lab owns 2 scFv phage display selection libraries. After four selection rounds and a primary screening by ELISA and sequence analysis, the clones obtained are summarized in **Table Sn2**. For the GFS-5 peptide selection, 368 clones were picked. Based on ELISA screening data, 105 clones were sequenced. Of these 29 clones were unique and among them 13 clones were able to bind to the native type II collagen. The primary ELISA and HTRF screen data of 77 clones are shown in **Table S1** including binding to GFS-5, GFS-15, GFS-2, native and denatured type II collagen, and streptavidin control. Twenty-nine out of the 77 clones showed binding to native collagen. Twenty-three binders that are specific for native collagen were selected.

Table. Sn2. Phage display selection summary from three peptides.

Target	GFS-5	GFS-15	GFS-2
Clones screened	368	368	184
Clones sequenced	105	160	67
Unique clones	29	48	42
Clones binding native Collagen II	13	10	6

Primary screening by Luminex

Both anti-FLAG-tag (R-PE) and anti-His-tag conjugated antibodies were used. Between the 2 detection antibodies, the signal to noise of the anti-FLAG tag antibody is significantly higher. Among the 77 unique clones, 25 clones showed specific binding to GFS-5 (COL2F4 R-R) and GFS-15 (COL2F4 Cit-R) peptides as well as to native collagen. The data have been compared with initial ELISA data and they correlate well despite some differences in their binding preferences. The differences in relative signal intensity could be expected based on method differences; one is solid phase and the other, more or less in solution. Also, scFv proteins from two preparations have been used. In addition, one clone showed cross-reactivity with the cyclic COL2F4 (R-R) peptide, and four clones showed cross-reactivity with non-COL2F4 epitopes.

Biacore analysis

The binding kinetics was analyzed by Biacore and the results are summarized in **Table. Sn3**. Representative sensorgrams are shown in **Fig. Sn2**. The overall binding affinity to native collagen was high, up to 10^9. The two non-binders neither showed binding to native collagen as analyzed by ELISA.

Table. Sn3. Thermodynamic (KD) and kinetic (Kon and Koff) parameters of scFv binding to COL2 as determined by single-cycle kinetics.

Ligand	Epitope	ka (1/Ms)	kd (1/s)	KD (M)	Chi^2 (RU^2)
**scFv-36**	COL2 F4	1.9E+06	1.3E-05	6.9E-12	0.008
**scFv-49**	COL2 F4	1.6E+06	1.3E-03	8.3E-10	0.009
**scFv-47**	COL2 F4	1.8E+06	1.7E-03	9.5E-10	0.008
**scFv-23**	COL2 F4	5.9E+05	1.0E-03	1.7E-09	4.950
**scFv-41**	COL2 F4	2.9E+05	1.0E-03	3.4E-09	1.090
**scFv-25**	COL2 F4	7.8E+05	2.8E-03	3.6E-09	0.038
**scFv-21**	COL2 F4	3.7E+05	1.4E-03	3.8E-09	0.485
**scFv-29**	COL2 F4	4.1E+05	1.7E-03	4.0E-09	1.510
**scFv-30**	COL2 F4	2.1E+06	8.8E-03	4.3E-09	0.021
**scFv-56**	COL2 F4	5.9E+05	2.8E-03	4.7E-09	0.106
**scFv-53**	COL2 F4	4.1E+05	2.0E-03	4.8E-09	0.597
**scFv-26**	COL2 F4	4.1E+05	2.3E-03	5.6E-09	0.482
**scFv-7**	COL2 F4	4.3E+05	2.4E-03	5.7E-09	0.372
**scFv-4**	COL2 F4	2.8E+05	1.6E-03	5.7E-09	0.145
**scFv-34**	COL2 F4	3.0E+05	1.9E-03	6.2E-09	0.046
**scFv-60**	COL2 F4	4.1E+05	2.6E-03	6.5E-09	0.242
**scFv-20**	COL2 F4	2.9E+05	1.9E-03	6.6E-09	0.174
**scFv-57**	COL2 F4	5.9E+05	4.2E-03	7.1E-09	0.172
**scFv-28**	COL2 F4	6.5E+05	4.7E-03	7.3E-09	0.333
**scFv-46**	COL2 F4	5.9E+05	4.8E-03	8.3E-09	0.591
**scFv-24**	COL2 F4	3.0E+05	2.5E-03	8.3E-09	0.276
**scFv-18**	COL2 F4	4.5E+05	3.8E-03	8.4E-09	2.190
**scFv-2**	COL2 F4	2.7E+05	2.5E-03	9.3E-09	0.185
**scFv-19**	COL2 F4	1.8E+10	2.1E+02	1.1E-08	0.036
**scFv-14**	COL2 F4	3.1E+05	4.4E-03	1.4E-08	1.260
**scFv-50**	COL2 F4	3.7E+04	1.3E-03	3.6E-08	0.003
**scFv-69**	COL2 F4	"No binding detected"			
**scFv-70**	COL2 F4	"No binding detected"			

Fig Sn2. Representative Biacore sensorgrams processed using automatic correction for nonspecific bulk-refractive-index effects.

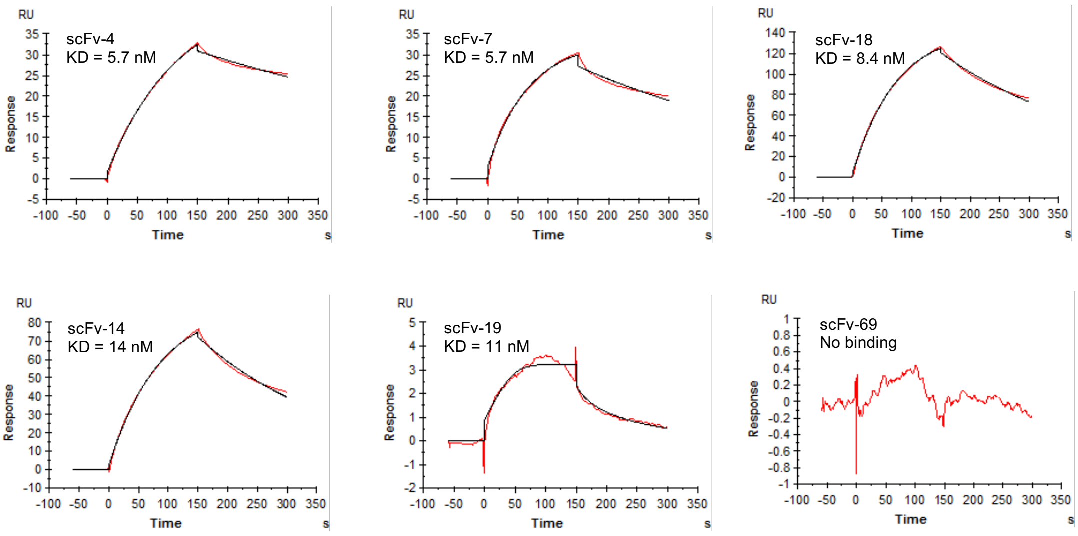

Joint tissue staining

Binding of scFv's to neonatal frozen tissue was done using biotin labelled anti-His Ab and Avidin peroxidase. Binding was observed for 17 scFv's and categorized as Strong, Mild and Weak, respectively depending on the color intensity. Thirteen scFv's did not show any binding. The staining was summarized in **Table S2**, and representative images were shown in **Fig. Sn3**.

Fig Sn3. Representative IHC staining of neonatal cartilage.

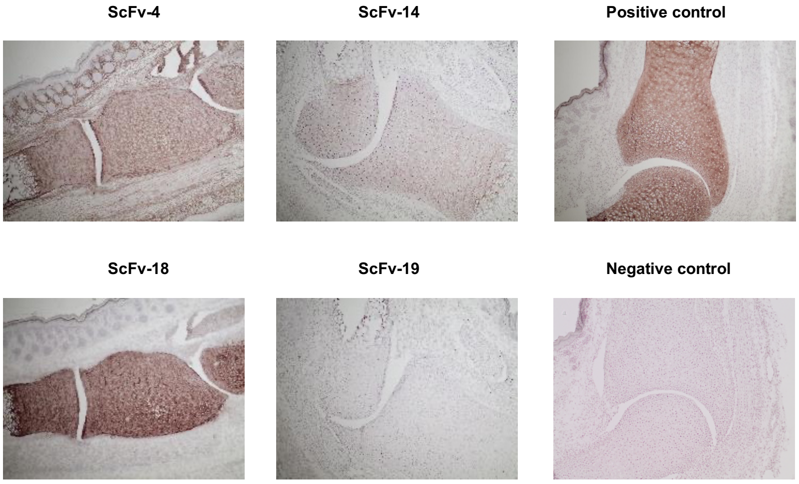

Selection of binders for full-length conversion

Four binders were selected for an initial round of conversion to full-length based on the following criteria: 1) Positive in binding to neonatal tissue; 2) Positive in binding to synthetic 24-aa COL2F4 triple helix peptide; 3) Positive in binding to recombinant 24-aa COL2F4 triple helix peptide and 4) Positive in binding to synthetic 12-aa COL2F4 triple helix peptide. Those clones are scFv-14, -18, -28 and -60.

In addition, scFv-4 was selected because of its high binding to neonatal cartilage; scFv-7 was included because of lack of binding to any version of the COL2F4 peptides therefore serving as a control; scFv-19 was included because it does not bind to neonatal cartilage tissue, therefore was also used as a control.

Full-length antibody expression and purification

The expression level varied from 18 to 187 mg/L. The quality of the R69-60 antibody as analyzed by SDS-PAGE under reducing conditions was inferior also after repeating the experiment. It was therefore excluded. R69-28 was also excluded because its expression was too low to support animal experiment. Thus, 5 antibodies remained and were subject to large scale production. Those clones are R69-4, -7, -14, -18, and -19.

CAIA experiment

Results shown in **Figure S1a.**
