# Supplementary material for: A subset of type-II collagen-binding antibodies prevents experimental arthritis by inhibiting FCGR3 signaling in neutrophils
Source: Nat Commun. 2023 Sep 23;14:5949. doi: 10.1038/s41467-023-41561-7 (PMC10517938; doi:10.1038/s41467-023-41561-7)
Supplement: Supplementary file 3 — Reporting Summary [file 41467_2023_41561_MOESM3_ESM.pdf]

nature portfolio

Corresponding author(s): Rikard Holmdahl

Last updated by author(s): Aug 22, 2023

Reporting Summary

Nature Portfolio wishes to improve the reproducibility of the work that we publish. This form provides structure for consistency and transparency in reporting. For further information on Nature Portfolio policies, see our Editorial Policies and the Editorial Policy Checklist.

Statistics

For all statistical analyses, confirm that the following items are present in the figure legend, table legend, main text, or Methods section.

n/a	Confirmed	
☐	☒	The exact sample size (*n*) for each experimental group/condition, given as a discrete number and unit of measurement
☐	☒	A statement on whether measurements were taken from distinct samples or whether the same sample was measured repeatedly
☐	☒	The statistical test(s) used AND whether they are one- or two-sided<br>*Only common tests should be described solely by name; describe more complex techniques in the Methods section.*
☒	☐	A description of all covariates tested
☐	☒	A description of any assumptions or corrections, such as tests of normality and adjustment for multiple comparisons
☐	☒	A full description of the statistical parameters including central tendency (e.g. means) or other basic estimates (e.g. regression coefficient) AND variation (e.g. standard deviation) or associated estimates of uncertainty (e.g. confidence intervals)
☐	☒	For null hypothesis testing, the test statistic (e.g. *F*, *t*, *r*) with confidence intervals, effect sizes, degrees of freedom and *P* value noted<br>*Give P values as exact values whenever suitable.*
☒	☐	For Bayesian analysis, information on the choice of priors and Markov chain Monte Carlo settings
☒	☐	For hierarchical and complex designs, identification of the appropriate level for tests and full reporting of outcomes
☒	☐	Estimates of effect sizes (e.g. Cohen's *d*, Pearson's *r*), indicating how they were calculated

Our web collection on statistics for biologists contains articles on many of the points above.

Software and code

Policy information about availability of computer code

Data collection	Gen5 v1.05, Attune v5.2.0, Bio-rad CFX Manager v3.1, Zen blue v3.1
Data analysis	Microsoft Office 16, GraphPad Prism v9.5.0, MaxQuant v2.4.2.0, FlowJo v10.8.1

For manuscripts utilizing custom algorithms or software that are central to the research but not yet described in published literature, software must be made available to editors and reviewers. We strongly encourage code deposition in a community repository (e.g. GitHub). See the Nature Portfolio guidelines for submitting code & software for further information.

Data

Policy information about availability of data

All manuscripts must include a data availability statement. This statement should provide the following information, where applicable:

- Accession codes, unique identifiers, or web links for publicly available datasets
- A description of any restrictions on data availability
- For clinical datasets or third party data, please ensure that the statement adheres to our policy

The source data supporting the findings of this study are available within the paper (Supplementary Information). The mass spectrometry proteomics data have been deposited to the ProteomeXchange Consortium via the PRIDE partner repository with the dataset identifier PXD044700. All other data and the unique reagents used in this study are available from the corresponding author upon reasonable request.

Research involving human participants, their data, or biological material

Policy information about studies with human participants or human data. See also policy information about sex, gender (identity/presentation), and sexual orientation and race, ethnicity and racism.

Reporting on sex and gender	N/A
Reporting on race, ethnicity, or other socially relevant groupings	N/A
Population characteristics	N/A
Recruitment	N/A
Ethics oversight	N/A

Note that full information on the approval of the study protocol must also be provided in the manuscript.

Field-specific reporting

Please select the one below that is the best fit for your research. If you are not sure, read the appropriate sections before making your selection.

☒ Life sciences ☐ Behavioural & social sciences ☐ Ecological, evolutionary & environmental sciences

For a reference copy of the document with all sections, see nature.com/documents/nr-reporting-summary-flat.pdf

Life sciences study design

All studies must disclose on these points even when the disclosure is negative.

Sample size	Sample sizes were determined according to the 3Rs principles (Replace, Refine and Reduce) in which minimum numbers of mice are suggested when animal experiments are necessary. The minimum numbers of animals chosen should warrant statistical analyses with sufficient power. Briefly, in animal models with 100% of incidence (e.g., CAIA), minimum 4 mice were included in each group; in animal models with relatively high but not 100% of incidence (e.g., GPIA), minimum 6 mice were included in each group. Sample sizes were disclosed in each experiment.
Data exclusions	No exclusion.
Replication	Key experiments were replicated at least twice using identical protocols.
Randomization	Animals were randomly allocated to different groups, with littermates control strategy applied.
Blinding	All scoring procedures were carried out blindly for animal experiments. In other experiments, blinding strategy was applied when subjective evaluation was required, e.g., confocal imaging.

Reporting for specific materials, systems and methods

We require information from authors about some types of materials, experimental systems and methods used in many studies. Here, indicate whether each material, system or method listed is relevant to your study. If you are not sure if a list item applies to your research, read the appropriate section before selecting a response.

Materials & experimental systems

n/a	Involved in the study	
☐	☒	Antibodies
☒	☐	Eukaryotic cell lines
☒	☐	Palaeontology and archaeology
☐	☒	Animals and other organisms
☒	☐	Clinical data
☒	☐	Dual use research of concern
☒	☐	Plants

Methods

n/a	Involved in the study	
☒	☐	ChIP-seq
☐	☒	Flow cytometry
☒	☐	MRI-based neuroimaging

Antibodies

Antibodies used	Hybridoma produced antibodies: M2139, ACC1, 15A, L10D9, 2.4G2<br>Recombinant antibodies: R69 series<br>Commercial antibodies: PE/Cy7-anti-CD16/32 (93, BioLegend); PE/Cy7-anti-CD16 (S17014E, BioLegend); FITC-anti-CD16 (S17014E, BioLegend), APC-anti-CD16.2 (9E9, BioLegend), Pacific blue-anti-CD45R (RA3-6B2, BD Biosciences), PerCP/Cy5.5-anti-CD3ε (500A2, BioLegend), Pacific blue-anti-CD11b (M1/70, BioLegend), FITC-anti-CD11b (M1/70, BD Biosciences), BV605-anti-Ly-6C (HK1.4, BioLegend), PE-anti-Ly-6G (1A8, BioLegend), FITC-anti-Ly-6G (1A8, BioLegend), FITC-anti-NK1.1 (PK136, BioLegend), FITC-anti-CD18 (M18/2, BioLegend), APC-anti-CXCR2 (SA044G4, BioLegend), PerCP/Cy5.5-anti-CXCR4 (L276F12, BioLegend), PE/Cy7-anti-C5aR (20/70, BioLegend), PE-anti-Igλ (RML-42, BioLegend), APC-anti-phosphotyrosine (PY20, BioLegend), PE anti-PLCγ2 Phospho (Tyr759) (QA20A56, BioLegend), APC-anti-F4/80 (BM8, BioLegend), FITC-anti-C1q (JL-1, Nordic Biosite), FITC-anti-Igκ (1050-02, Southern Biotech), AF488-anti-IgG2b (polyclonal, ThermoFisher Scientific), anti-human IL-34 (E0320E7, BioLegend).
Validation	The arthritogenicity of the antibody cocktail was validated by pilot animal experiments using FCGR2B- mice. The validation of Fc-blocking antibodies was performed by culturing pre-blocked cells and specific fluorescence conjugated antibodies against FCGRs. The commercial flow-cytometry antibodies were validated by the successful separation of the given positive and negative populations, as well as by referring to the the validation statements from manufactures.

Animals and other research organisms

Policy information about studies involving animals; ARRIVE guidelines recommended for reporting animal research, and Sex and Gender in Research

Laboratory animals	Mouse strains used in this study: BQ.Fcgr2b-, BQ.Ncf1*, BQ.Cia9i, BQ, BQ.Fcgr2b-.Hc*,BQ.Fcgr3-. All animals were aged 10-16 weeks when put into experiments. Animals were housed under FELASA2 specific pathogen-free (SPF) conditions.
Wild animals	None.
Reporting on sex	All animals included in this study were males.
Field-collected samples	None.
Ethics oversight	All experiments were carried out according to the ethical permits (No: N35/16 and 2660-2021) approved by the animal ethics committee at the Swedish Board of Agriculture.

Note that full information on the approval of the study protocol must also be provided in the manuscript.

Flow Cytometry

Plots

Confirm that:

☒ The axis labels state the marker and fluorochrome used (e.g. CD4-FITC).

☒ The axis scales are clearly visible. Include numbers along axes only for bottom left plot of group (a 'group' is an analysis of identical markers).

☒ All plots are contour plots with outliers or pseudocolor plots.

☒ A numerical value for number of cells or percentage (with statistics) is provided.

Methodology

Sample preparation	Single-cell suspensions were prepared from mouse PB, SF, or BM samples. For peripheral blood mononuclear cells (PBMCs) preparation, 30 µL of venous blood were collected by cheek bleeding and buffered in PBS containing heparin on ice. Red blood cells (RBCs) were lysed using 1mL of ammonium-chloride-potassium (ACK) buffer. SF samples were taken from bilateral knees after termination and buffered in PBS, and BM samples were taken by flushing BM cavity of left femur with PBS. Cells from both SF and BM were filtered using 70-µm cell strainers (BD Biosciences) to remove tissues.<br>Single cells were pre-incubated with Live/Dead fixable Near-IR dye (ThermoFisher Scientific) plus Fc-block or fluorescent antibody against FCGRs (anti-CD16/32 (2.4G2); PE/Cy7-anti-CD16/32 (93, BioLegend); PE/Cy7-anti-CD16 (S17014E, BioLegend); FITC-anti-CD16 (S17014E, BioLegend), and/or APC-anti-CD16.2 (9E9, BioLegend)) for 20 min at room temperature. Next, the cells were stained with 50 µL of master mix containing all staining antibodies for 30 min at room temperature.<br>In terms of intracellular staining, cells were fixed by 100 µL of Cytofix/CytoPerm solution (BD Biosciences) for 30 min, and the intracellular targets were stained with corresponding antibodies diluted in Perm/wash buffer (BD Biosciences) for 1 h. After the staining, cells were washed 3 times using the same Perm/wash buffer prior to flow cytometry analysis.
Instrument	Attune NxT flow cytometer
Software	Attune Cytometric Software v5.2.0

Cell population abundance	No sorting carried out
Gating strategy	1. White blood cells were gated based on FSC-A and SSC-A features;<br>2. Single cells were gated based on FSC-A and FSC-H features;<br>3. Live cells were gated based on Live/Dead NIR negative staining;<br>4. Myeloid cells (plus NK cells) were gated based on CD11b positive staining;<br>5. Neutrophils were gated based on high Ly-6G staining and medium Ly-6C staining;<br>6. Monocytes were gated based on high Ly-6C staining and negative Ly-6G staining.<br>Boundaries of the gates were determined by the visualization of distinct populations or comparing samples and positive/ negative controls.

☒ Tick this box to confirm that a figure exemplifying the gating strategy is provided in the Supplementary Information.
